# Supplementary material for: Is Self-Reported Physical Fitness Useful for Estimating Fitness Levels in Children and Adolescents? A Reliability and Validity Study
Source: Medicina (Kaunas). 2019 Jun 18;55(6):286. doi: 10.3390/medicina55060286 (PMC6631242; doi:10.3390/medicina55060286)
Supplement: Supplementary file 1 [file medicina-55-00286-s001.pdf]

medicina

Supplementary material

Table S1. Means of performance by physical fitness tests in children (3 to 10 years).

Measured physical fitness	Low/poor (mean)	Good/Great (mean)
Cardiorespiratory fitness: 20-m shuttle run (stage)	2.5	4.4
Muscular fitness: Handgrip (kg)	6.4	14.2
Standing long jump (cm)	63.8	116.9
Speed-Agility: Shuttle run 4 × 10 m (s)*	22.6	14.1
Flexibility: Sit and reach (cm)	19.3	30.6

* The lower the score (time in seconds) the better the performance.

Table S2. Means of performance by physical fitness tests in adolescent (11 to 17 years).

Measured physical fitness	Very poor (mean)	Poor (mean)	Average (mean)	Good (mean)	Very good (mean)
Cardiorespiratory fitness 20-m shuttle run (stage)	3.2	3.6	4.3	5.3	6.7
Muscular fitness Handgrip (kg)	25.5	27.2	29.3	32.0	35.5
Standing long jump (cm)	148.1	151.7	158.4	170.2	190.1
Speed-Agility Shuttle run 4 × 10 m (s)*	13.3	12.6	12.0	11.8	10.6
Flexibility Sit and reach (cm)	15.4	18.1	21.3	24.4	27.5

* The lower the score (time in seconds) the better the performance.
